# Effect of ruxolitinib on the oral mucosa of patients with steroid-refractory chronic Graft-versus-Host disease and oral involvement

**DOI:** 10.1007/s00784-022-04393-1

**Published:** 2022-02-16

**Authors:** Martina Kaurinovic, Konstantina Delli, Ana-Mae E. Jonk, Anouschka Biswana, Carin L. E. Hazenberg, Goda Choi, Marco R. de Groot, Linde M. Morsink, Arjan Vissink, Mar Bellido

**Affiliations:** 1 Department of Oral and Maxillofacial Surgery, University Medical Center Groningen, University of Groningen, Hanzeplein 1, 9713 GZ Groningen, the Netherlands; 2 Department of Hematology, University Medical Center Groningen, University of Groningen, Hanzeplein 1, 9713 GZ Groningen, the Netherlands

**Keywords:** Graft vs host disease, Mouth mucosa, INCB018424, Immunosuppression

## Abstract

**Background:**

Chronic Graft-versus-Host Disease (cGVHD) can impact quality of life, especially in patients with oral involvement. Half of the patients with cGVHD do not respond to first-line therapy with corticosteroids and calcineurin inhibitors. Ruxolitinib is effective in steroid-refractory (SR)-cGVHD cases, but the long-term effects of ruxolitinib on the oral mucosa are unknown.

**Objective(s):**

This study aims to assess the effect of ruxolitinib on the oral mucosa of SR-cGVHD patients with oral involvement.

**Materials and methods:**

An observational longitudinal patient study was conducted in 53 patients with SR-cGVHD and oral involvement who were treated with ruxolitinib. The baseline condition of the oral mucosa was compared to its condition at 4 and 12 weeks after starting ruxolitinib.

**Results:**

The overall response was 81% (43/53), with a complete response in 53% (28/53) and partial response in 28% (15/53) after 12 weeks (*p* < 0.001). Men and patients concurrently using immunosuppressive therapy responded better than women (*p* = 0.005) and patients with ruxolitinib monotherapy (*p* = 0.02), respectively. At a longer follow-up (median 20 months), oral symptoms were comparable to the 12-week symptoms (*p* = 0.78), regardless of ruxolitinib use (*p* = 0.83).

**Conclusion:**

Ruxolitinib treatment of SR-cGVHD patients with oral involvement was associated with a significant response of the oral manifestations at 12 weeks.

**Clinical relevance:**

The oral mucosa of SR-cGVHD patients is likely to improve after 4 and 12 weeks of ruxolitinib treatment. Symptom severity at baseline does not affect the response of the oral mucosa.

**Supplementary Information:**

The online version contains supplementary material available at 10.1007/s00784-022-04393-1.

## Introduction

Oral involvement in patients with chronic Graft-versus-Host Disease (cGVHD) after allogeneic hematopoietic stem cell transplantation is a prevalent complication which limits the quality of life of survivors [1, 2]. Oral mucosal involvement is seen in 45–83% of patients with cGVHD and consists of reticular patterns, erythema, pseudomembranes and/or ulcerations [1]. Oral manifestations of cGVHD can be distinguished from radio- and chemotherapy-induced oral mucositis by the moment of symptom onset and pathogeny. Radio- and chemotherapy-induced mucositis in patients who received an allogeneic stem cell transplant appears shortly after the start of these treatments and resolves after the neutrophilic recovery, usually, within 4 weeks after treatment [3, 4]. Alterations of the oral mucosa due to cGVHD appear during repopulation, often several months after the allogeneic stem cell transplantation [4]. Although tissue damage and inflammation are seen in both chemo- and radiotherapy-induced oral mucositis and oral manifestations of cGVHD, the pathomechanism differs [4, 5]. Chemo- and radiotherapy-induced tissue damage due to the release of free radicals and damage to DNA result in apoptosis of the epithelial cells [5]. The pathomechanism of cGVHD starts with inflammation triggering the innate immune response, followed by chronic inflammation triggering B- and T-cell populations with alloimmune reactions, and finally by altered macrophage polarization and aberrant tissue repair leading to fibrosis [6–8]. This is the reason that oral manifestations of cGVHD appear later than radio- and chemotherapy-induced mucositis. In clinical practice, oral mucositis of cGVHD is easily distinguished from oral mucositis induced by radio-chemotherapy.

Approximately half of the patients do not respond to first line treatment [9–11] with topical or systemic corticosteroids, resulting in steroid-refractory (SR)-cGVHD. These patients are candidates for second line treatment [12]. Ruxolitinib, a Janus kinase 1 (JAK1) and JAK2 inhibitor, has been shown to be effective in the treatment of several organs affected by steroid refractory (SR)-cGVHD [13–22]. Although oral lesions are a major manifestation of SR-cGVHD, the effect of ruxolitinib on the oral mucosa has sparsely been reported. Therefore, the aim of the current study was to assess the efficacy of ruxolitinib in reducing symptoms and manifestations of oral cGVHD in SR-cGVHD patients.

## Materials and methods

This study is an observational, retrospective, longitudinal study where patients were prospectively followed-up. For the retrospective data collection, the oral mucosa at baseline was compared to the oral mucosa at 4 and 12 weeks after initiation of ruxolitinib treatment as reported in patient records. For patients included in the analysis treatment with ruxolitinib was started at the Department of Hematology of the University Medical Center Groningen (UMCG) between January 2014 and October 2019. For the prospective data collection, patients were recalled for a follow-up visit between February 2020 and March 2020 to investigate the long-term effects of ruxolitinib on the oral mucosa. Data were collected under approval of the Medical Ethics Review Board of the UMCG (M19.232532).

### Study population

Patients were included if they had oral manifestations of SR-cGVHD with involvement of cGVHD of other organs; or had oral manifestations of SR-cGVHD with involvement of acute GVHD of other organs; and were 18 years or older. The excluded patients had received ruxolitinib for indications other than SR-cGVHD. SR-cGVHD was defined by Schoemans et al. [23]. Patients meeting the inclusion criteria had previously been treated with oral prednisolone and a calcineurin inhibitor (cyclosporin or tacrolimus), and for the oral manifestations with topical dexamethasone, triamcinolone or clobetasol. Ruxolitinib was added because of refractoriness to first-line treatment. Patients were treated with either 5 mg or 10 mg ruxolitinib orally administered twice daily, depending on the severity of the cGVHD. Ruxolitinib doses were modified adding or stepping down 5 mg according to published results [24].

### Variables and methods

The severity of oral cGVHD of patients in whom ruxolitinib treatment was started, was routinely scored using the *NIH Mouth Staging Score* (NIH-MSS) [25] (score range 0–3), with score 0 being assigned for no symptoms and score 3 for severe symptoms with large limitation of oral intake. This scoring was done in all patients at three moments, i.e. before starting the ruxolitinib (baseline), and at 4 and 12 weeks after the start of treatment. The scores were extracted from patient records.

For the follow-up assessment, the latest NIH-MSS data were extracted from the patient records as well as obtained by oral mucosa inspection of the patients who visited the clinic between February and March 2020 for a routine follow-up. The oral mucosa was also scored using a number of validated scales [11], viz., *NIH modified Oral Mucosal Rating Scale* (erythema, lichen-like lesions and ulcers: 0–3) [26], *Escudier Scale* (size and severity: 0–24, activity: 0–72) [27], *Johns Hopkins Mouth Pain score* (0–3) [28], *NIH Oral Symptom Score* (mouth dryness, pain and sensitivity: 0–10) [29] and *Lee cGVHD Symptom Scale* (seven items, 0–4) [30]. For all scales, a score of 0 was assigned in the absence of symptoms and the maximum score was given for severe symptoms.

The guidelines of the NIH consensus criteria from 2014 were followed to classify complete response (CR) and partial response (PR) [26]. CR was defined as complete disappearance of disease symptoms and PR for an improvement of the score by at least 1 point on a 4-point scale. CR and PR combined formed the overall response rate (ORR). It is important to note that the CR, PR and ORR in this study related to the mouth only and no statement was made about response in other organs. Patients who started immunosuppressive therapy during the study period could not be scored as CR or PR and therefore were not included in the ORR [26].

To ensure anonymity, patients were given a Unique Patient Number. Data was collected in Microsoft Excel v.16.36 2020 (Microsoft Corporation, Redmond, New York, USA) and stored in the digital environment of the UMCG.

### Statistical analysis

Results were analyzed using IBM SPSS Statistics v.26 2019 (IBM, Armonk, New York, USA). *P*-value < 0.05 was considered statistically significant [31]. A Friedman test was performed, followed by the Wilcoxon signed-rank test with a Bonferroni correction as post-hoc analysis. Furthermore, a statistical model was built with the Generalized Estimating Equations to analyze the efficacy of ruxolitinib after 4 and 12 weeks. Confounders considered were gender, age, ruxolitinib dose, immunosuppressive therapy, topical therapy and score at baseline. Different correlation structures (exchangeable, M-dependent, unstructured) were tested and the model with the lowest information criterion was used, which was the exchangeable correlation structure for all variables.

## Results

**Fig. 1:**
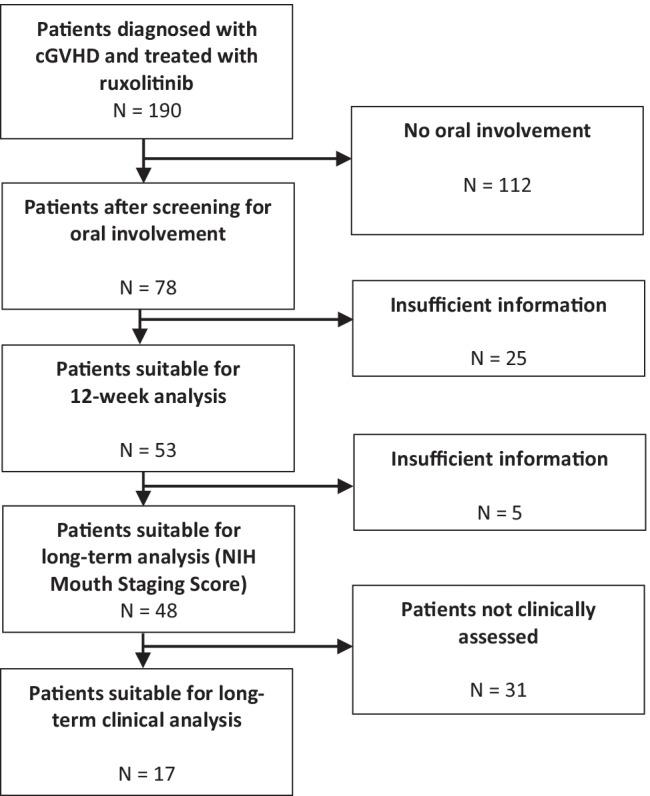
Flowchart showing the patient selection

Between 2014 and 2019, 190 patients had been treated with ruxolitinib for cGVHD, of whom 78 patients had oral manifestations of cGVHD (Fig. 1). A total of 53 patients were included (Table 1). At follow-up (median 20 months, range 2–56 months), the NIH-MSS was available for 48 patients, of whom 17 patients were seen for a routine follow-up in February and March 2020 (median 26 months, range 7–56 months) and underwent an extensive screening of their oral mucosa using all validated scales (Supplementary Table 1).

**Table 1:** Characteristics of the included patients

Characteristic	*n*	%
Patients	53	100
Gender		
Male	29	55
Female	24	45
Age (years)		
Mean	60	
Range	26–76	
Total body irradiation		
No	3	6
2 Gy	47	89
4 Gy	1	2
12 Gy	2	4
Overall GVHD severity		
Mild	10	19
Moderate	35	66
Severe	8	15
Organ involvement		
Skin (chronic)	31	58
Skin (acute)	4	8
Eyes (chronic)	23	43
Gastro-intestinal Tract (chronic)	3	6
Gastro-intestinal Tract (acute)	1	2
Liver (chronic)	8	15
Lungs (chronic)	5	9
Joints and Fascia (chronic)	5	9
Genital Tract (chronic)	4	8
Ruxolitinib dose		
5 mg b.i.d	30	57
10 mg b.i.d	23	43
Immunosuppressive therapy during research period		
Yes	48	91
No	5	9
Initiation of immunosuppressive therapy during research period		
Yes	1	2
No	52	98
Topical treatment of any type during research period		
Yes	24	45
No	29	55
Side effects		
No	45	85
Hospitalization	5	9
Infection	3	6

### Oral manifestations (after 4 and 12 weeks)

**Fig. 2:**
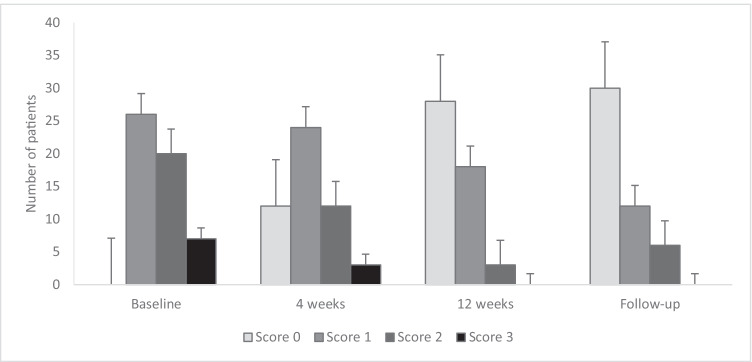
Presentation of the score distribution on the NIH Mouth Staging Score at baseline (*N* = 53), at 4 weeks (*N* = 51), at 12 weeks (*N* = 49) and at follow-up (*N* = 48). Scores range from 0 to 3, representing no symptoms (score 0), mild symptoms without limitation of oral intake (score 1), moderate symptoms with partial limitation of oral intake (score 2) and severe symptoms with limitation of oral intake (score 3). Error bars represent the standard error of mean

Oral manifestations of cGVHD were found to have decreased significantly at 4 weeks (median NIH-MSS = 1, *p* = 0.002) and 12 weeks (median NIH-MSS = 0, *p* < 0.001) after initiation of treatment (median NIH-MSS = 2) (Fig. 2). The ORR after 4 and 12 weeks of treatment was 51% and 81%, respectively. After 4 and 12 weeks, CR was achieved in 11 (21%) and 28 (53%) patients, PR in 16 (30%) and in 15 (28%) patients and symptoms remained stable in 19 (36%) and 6 (11%) patients, respectively.

As shown in Table 2, male patients and patients who received systemic immunosuppressive therapy responded better to ruxolitinib than female patients (*p* = 0.005) and patients not receiving systemic immunosuppressive therapy (*p* = 0.02). Men and women did not differ in history of total body irradiation (TBI), use of immunosuppressive therapy or topical therapy. Patients whose systemic immunosuppressant dose was increased due to worsening of cGVHD (*n* = 7, Table 3), had NIH-MSS scores that were comparable to patients whose dose was not increased and were therefore not excluded from the analysis. Prednisolone, tacrolimus and cyclosporin doses could be reduced in 59%, 35% and 18% of users, respectively (Table 3).

**Table 2:** Generalized Estimating Equations Model with undermentioned factors as potential predictors of the treatment outcome based on the NIH Mouth Staging Score during the 12-week study period. Scores of the NIH Mouth Staging Score range from 0 to 3, representing no symptoms (0) and severe symptoms with limitation of oral intake (3), respectively. *N* = 53

	B^a^	*p*
Gender ^0 = men, 1 = women^	0.38	0.005*
Age	− 0.01	0.35
Ruxolitinib dose	− 0.01	0.65
Immunosuppressive therapy during research period ^0 = no, 1 = yes^	− 0.46	0.02*
Topical treatment during research period ^0 = no, 1 = yes^	0.15	0.25
Visit	− 0.41	< 0.001*
Score at baseline	0.03	0.71

^*a*^ B represents the multiplier factor on the NIH Mouth Staging Score. * Statistically significant (p < *0.05)*

**Table 3:** Dose alteration and number of users of the used systemic immunosuppressants during the 12-week study period and at longer follow-up

		12 weeks		Follow-up	
Systemic immuno-suppressant		n users (%)	Dose change in mg/day, median (range)	n users (%)	Dose change in mg/day, median (range)
Prednisolone	Total	39 (100)		39 (100)	
	Reduction	23 (59)	20 (88)	30 (77)	20 (75)
	Increase	1 (3)	20 (0)	5 (13)	20 (23)
Tacrolimus	Total	20 (100)		19 (100)	
	Reduction	7 (35)	2 (2)	11 (58)	2 (3)
	Increase	4 (20)	2 (1)	3 (16)	1 (1)
Cyclosporin	Total	17 (100)		17 (100)	
	Reduction	3 (18)	100 (88)	13 (76)	100 (250)
	Increase	2 (12)	100 (100)	3 (18)	100 (50)

### Oral manifestations (longer follow-up)

At a longer follow-up (median 20 months, range 2–56 months), oral symptoms did not differ significantly from the oral symptoms experienced at 12 weeks (*p* = 0.78). Compared to the CR (53%) and PR (28%) at 12 weeks, the ORR at follow-up showed an increased number of conversions from PR (9%) to CR (57%). However, 13% (6/48) of patients experienced moderate symptoms with partial limitation of oral intake (NIH-MSS score 2) at follow-up compared to 6% (3/49) of patients at 12 weeks, indicating that some patients with score 1 probably converted to score 2 (Fig. 2). NIH-MSS score 2 was more prevalent for patients not using ruxolitinib than other patients, although ruxolitinib use at follow-up did not affect oral symptoms significantly (*p* = 0.83).

### Clinical evaluation (longer follow-up)

The clinical evaluation at follow-up (median 26 months, range 7–56 months) in February and March 2020 showed that 88% (15/17) of the patients had no or minor erythema, lichen-like lesions or ulcers (Supplementary Fig. 1). Lesions were mainly located on the buccal mucosa. In most cases, size and severity were limited to hyperkeratosis with mild erythema of < 50% of the area (Supplementary Fig. 2). Patients experienced mild symptoms according to *NIH Oral Symptom Scores* and 82% (14/17) avoided hardly any food according to *Lee cGVHD Symptom Scale*. A total of 12% (2/17) reported ulcers, 6% (1/17) experienced mild difficulty swallowing solid foods and 6% (1/17) of patients reported mild vomiting and weight loss.

## Discussion

This study shows that ruxolitinib is associated with a significant reduction of oral cGVHD in 81% of patients with SR-cGVHD and oral manifestations. Patients with mild, moderate and severe symptoms responded to ruxolitinib therapy, with a better response at 12 weeks than at 4 weeks. Women and patients not receiving immunosuppressive therapy responded less well than other patients, but nevertheless achieved satisfactory treatment results.

Within the period of the study, the maximal response was observed after 12 weeks of ruxolitinib treatment (ORR 81%). Therefore, in order to achieve optimal results ruxolitinib treatment should be continued for at least 12 weeks, even after initial signs of oral symptom improvement. The results from the follow-up section showed an ORR of 76%, which was rather comparable to the ORR after 12 weeks of ruxolitinib treatment. This result should be interpreted carefully as this is a heterogenous group with patients who differ in ruxolitinib use and treatment duration. Zeiser et al. [24] studied a more homogenous group of patients in which ruxolitinib was administered for a period of 24 weeks to assess the response of all organs. They found an ORR of 50% for the oral mucosa, less than the ORR of 76% in our study, which indicates that unlike other organs maximal response for the mouth could be achieved in less than 24 weeks.

Our study shows that patients with high NIH-MSS scores responded as well to ruxolitinib as patients with lower scores, regardless of possible differences in ruxolitinib dose (Table 2). This observation may be explained by the effect of ruxolitinib in the early as well as late phase of tissue damage in cGVHD [32]. Ruxolitinib inhibits JAK1 and JAK2, preventing inflammation and tissue damage [13, 32, 33], but ruxolitinib also inhibits chemokines causing final phase tissue damage [32].

The lower treatment responses of women and patients not receiving immunosuppressive therapy invite additional analyses. In our study, the male and female cGVHD patients treated with ruxolitinib were similar in terms of conditioning by TBI prior to transplantation, immunosuppressive therapy and topical treatment. The higher NIH-MSS scores in women might be associated with mouth sensitivity caused by hyposalivation, which is more intensely experienced by women [34]. However, Bassim et al. [35] could not find an association between the NIH-MSS and hyposalivation and concluded that salivary gland disease developed independently of cGHVD of the oral mucosa. In our study, patients who received immunosuppressive therapy responded better to ruxolitinib treatment than patients who did not. We hypothesize that a possible synergistic effect of ruxolitinib and other systemic immunosuppressive therapy may account for this difference, though this has not been reported in literature for other manifestations of cGVHD. Furthermore, the group on ruxolitinib monotherapy was small (*n* = 5) and treatment with ruxolitinib without other immunosuppressive therapy is not common, so this result should be interpreted with caution.

**Fig. 3:**
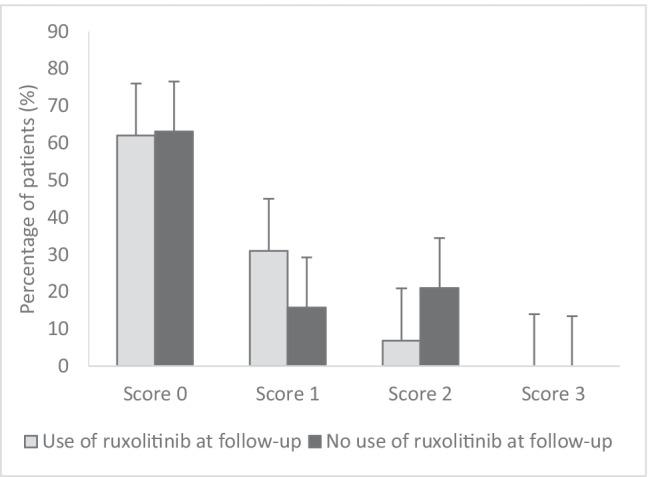
Score distribution of the NIH Mouth Staging Score of patients using ruxolitinib at follow-up (*N* = 29) and patients not using ruxolitinib at follow-up (*N* = 19). Scores range from 0 to 3, representing no symptoms (score 0), mild symptoms without limitation of oral intake (score 1), moderate symptoms with partial limitation of oral intake (score 2) and severe symptoms with limitation of oral intake (score 3). Error bars represent the standard error of mean

The long-term results showed that oral symptoms were comparable to the oral symptoms at 12 weeks, also for the 40% of SR-cGVHD patients in whom ruxolitinib treatment was ceased. However, the NIH-MSS showed an increase in patients with score 2 at follow-up. Patients not using ruxolitinib were overrepresented in this group (Fig. 3), suggesting that stopping ruxolitinib might lead to worsening of oral symptoms when present, which is illustrated by a shift from score 1 to score 2 on the NIH-MSS. Possibly, ruxolitinib can only be successfully discontinued for the mouth when disease symptoms completely resolve. The exact nature and the conditions to taper ruxolitinib remain to be determined.

Previous studies conducted by Zeiser et al. [24], Modi et al. [13], and Khoury et al. [16] found an ORR of 50%, 60% and 100%, respectively, for the mouth. The treatment duration varied from 6 months for the study of Zeiser et al. [24] and Modi et al. [13] to a median of 18 months for the study of Khoury et al. [16]. Our study showed that maximal response to ruxolitinib in the mouth may occur before 6 months and further ruxolitinib continuation does not translate in an improvement of the oral cGVHD.

This study is unique in several aspects. First of all, to date no study has enrolled such a large number of patients. Patients were systematically evaluated by a hematologist as well as oral medicine specialist, as recommended by Lee et al. [26]. In addition, possible confounding factors as gender and immunosuppressive therapy were considered in our study. Our study is also the first to demonstrate that oral mucosal symptoms and manifestations remained stable in the long-term and that ruxolitinib could successfully be discontinued in a number of patients.

Limitations are the observational nature of the study. Because the use and dosage of other medications was adapted over the 12-week period, the effect of ruxolitinib may have been over- or underestimated. According to the 2014 NIH consensus criteria [26], results from patients who also received topical agents should be interpreted with caution and analyzed for statistical differences. In this study, the application of topical agents did not appear to be a significant predictor of treatment outcome, even though patients who did not receive topical therapy had significantly lower NIH-MSS scores at baseline than patients who did. Patients who started immunosuppressive therapy during the study period were not included in the ORR as recommended by the 2014 NIH consensus criteria [26]. Our study used NIH-MSS, instead of the recommended *NIH modified Oral Mucosal Rating Scale* [26], because of availability in the patient records. Bassim et al. [36] showed that both scales are significantly correlated with each other. ORR determination based on the NIH-MSS is therefore feasible. Regarding the follow-up section, only the patients who visited the clinic between February and March 2020 were clinically assessed. Since patients that are in remission often have a longer recall term, this patient group may be underrepresented in our study. The positive results achieved with ruxolitinib raise the question whether ruxolitinib should replace corticosteroids as a first-line drug. It is not unlikely that such a treatment may even prevent further development of cGVHD when given at an early stage [32].

In conclusion, ruxolitinib is significantly associated with a sustainable amelioration of the oral symptoms in patients with oral involvement of SR-cGVHD who are treated 12 weeks with ruxolitinib, regardless of cGVHD severity. Future prospective studies should corroborate these findings.

## Supplementary Information

Below is the link to the electronic supplementary material.

Supplementary file1 (PDF 140 KB)
