# Supplementary material for: Effect of ruxolitinib on the oral mucosa of patients with steroid-refractory chronic Graft-versus-Host disease and oral involvement
Source: Clin Oral Investig. 2022 Feb 16;26(5):4209–16. doi: 10.1007/s00784-022-04393-1 (PMC9072523; doi:10.1007/s00784-022-04393-1)
Supplement: Supplementary file 1 — Supplementary file1 (PDF 140 KB) [file 784_2022_4393_MOESM1_ESM.pdf]

Supplementary Information (SI)

Title: Effect of Ruxolitinib on the Oral Mucosa of Patients with Steroid-Refractory Chronic Graft-versus-Host Disease and Oral Involvement

Journal: Clinical Oral Investigations

Authors: Martina Kaurinovic, Konstantina Delli, Ana-Mae E. Jonk, Anouschka Biswana, Carin L.E. Hazenberg, Goda Choi, Marco R. de Groot, Linde M. Morsink, Arjan Vissink

Corresponding Author: Martina Kaurinovic, University of Groningen, University Medical Center Groningen, Department of Oral and Maxillofacial Surgery, martina.kaurinovic@gmail.com

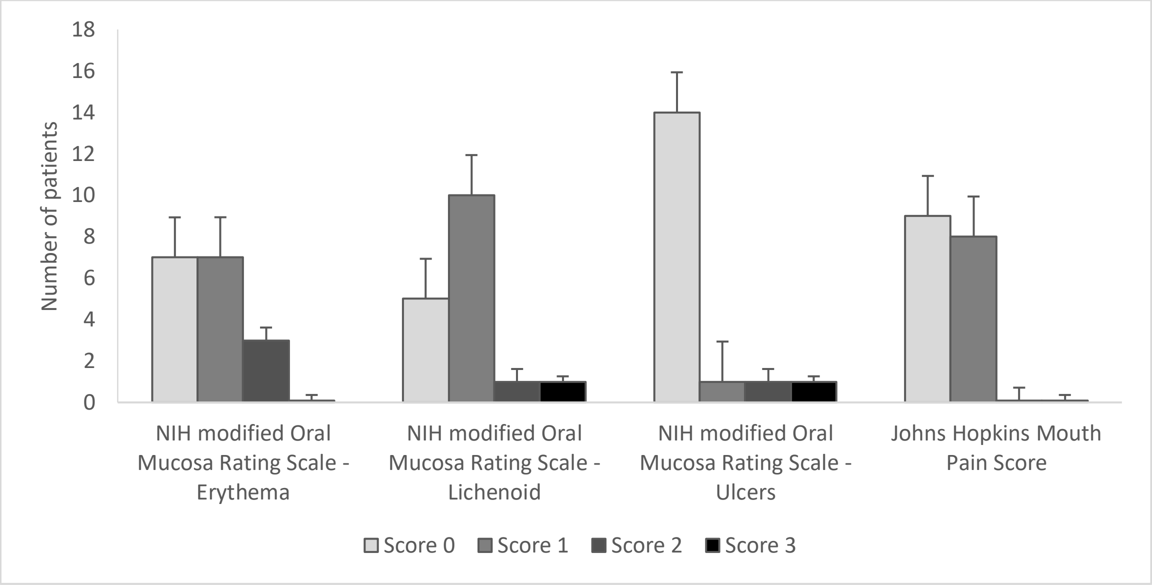

Supplementary Fig. 1 Distribution of the scores on several scales at longer follow-up (N=17). Scores range from 0 to 3 and error bars represent the standard error of mean. For the NIH modified Oral Mucosa Rating Scale – Erythema: score 0 = no symptoms; score 1 = mild erythema or moderate erythema (<25%); score 2 = moderate (>25%) or severe erythema (<25%); score 3 = severe erythema (>25%). For the NIH modified Oral Mucosa Rating Scale – Lichenoid: score 0 = no symptoms; score 1 = hyperkeratotic changes (<25%); score 2 = hyperkeratotic changes (25-50%); score 3 = hyperkeratotic changes (>50%). For the NIH modified Oral Mucosa Rating Scale – Ulcers: score 0 or 1 = no symptoms; score 2 = ulcers involving (<20%); score 3 = severe ulcerations (>20%). For the Johns Hopkins Mouth Pain Score: score 0 = no symptoms; score 1 = food sensitivity; score 2 = pain requiring narcotics; score 3 = unable to eat.

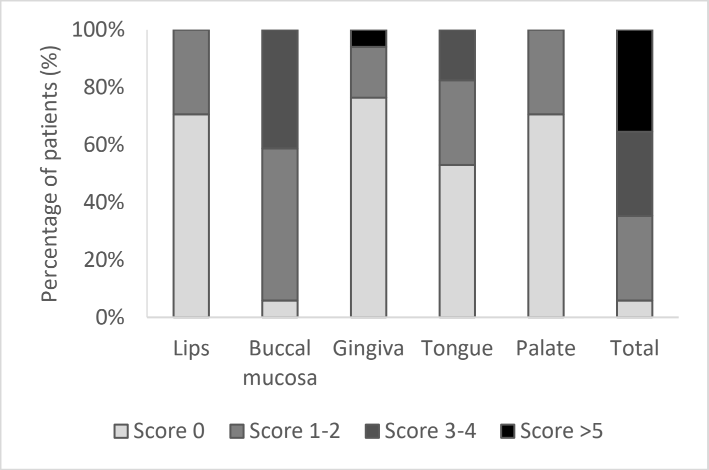

Supplementary Fig. 2 Score distribution of The Escudier Scale site score at follow-up (N=17). 0 represents no symptoms and >5 represents severe symptoms.

Supplementary Table 1 Characteristics of the patients in the longer follow-up (median 20 months, range 2-56 months) section.

		Total		Clinical assessment	
**Characteristics**		**n**	**%**	**n**	**%**
Patients		48	100	17	100
Gender					
	Male	26	54	9	53
	Female	22	46	8	47
Age (years)					
	Median	64		66	
	Range	26 – 76		26 – 75	
Follow-up in days from baseline					
	Median	621		803	
	Range	51 – 1703		217 – 1703	
Use of ruxolitinib at follow-up					
	Yes	29	60	5	71
	No	19	40	12	29
Use of ruxolitinib and/or immunosuppressive therapy (IT) at follow-up					
	Ruxolitinib and IT	18	38	3	18
	Only ruxolitinib	11	23	2	12
	Only IT	15	31	10	59
	No ruxolitinib nor IT	4	8	2	12
Topical treatment at follow-up					
	Yes	10	21	5	29
	No	38	79	12	71
